# A comparison of a social support physical activity intervention in weight management among post-partum Latinas

**DOI:** 10.1186/1471-2458-14-971

**Published:** 2014-09-19

**Authors:** Colleen Keller, Barbara Ainsworth, Kathryn Records, Michael Todd, Michael Belyea, Sonia Vega-López, Paska Permana, Dean Coonrod, Allison Nagle-Williams

**Affiliations:** Arizona State University, College of Nursing and Health Innovation, 500 N. 3rd Street; MC 3020, Phoenix, AZ 85004 USA; Arizona State University, School of Nutrition, Health Promotion, 500 N. 3rd Street; MC 3020, Phoenix, AZ 85004 USA; University of Missouri – Saint Louis, College of Nursing, 1 University Blvd, St. Louis, MO 63121 USA; Phoenix Veterans Affairs Health Care System, 650 E. Indian School Rd. Building 21, Room 147, Phoenix, AZ 85012 USA; Department of Obstetrics and Gynecology, Maricopa Integrated Health System/ District Medical Group, University of Arizona College of Medicine phoenix, 2525 East Roosevelt Street, Phoenix, AZ 85008 USA; Arizona State University, Southwest Interdisciplinary Research Center (SIRC), 411 N. Central Ave, Suite 720, Phoenix, AZ 85004 USA

**Keywords:** Latinas, Hispanics, Physical activity, Social support, Overweight, Obesity, Exercise

## Abstract

**Background:**

Weight gain during the childbearing years and failure to lose pregnancy weight after birth contribute to the development of obesity in postpartum Latinas.

**Methods:**

*Madres para la Salud* [Mothers for Health] was a 12-month, randomized controlled trial exploring a social support intervention with moderate-intensity physical activity (PA) seeking to effect changes in body fat, fat tissue inflammation, and depression symptoms in sedentary postpartum Latinas. This report describes the efficacy of the *Madres* intervention.

**Results:**

The results show that while social support increased during the active intervention delivery, it declined to pre-intervention levels by the end of the intervention. There were significant achievements in aerobic and total steps across the 12 months of the intervention, and declines in body adiposity assessed with bioelectric impedance.

**Conclusions:**

Social support from family and friends mediated increases in aerobic PA resulting in decrease in percent body fat.

**Trial registration:**

ClinicalTrials.gov Identifier: NCT01908959.

## Background

Childbearing age Latinas experience a heightened prevalence rate for obesity (45%) and overweight (76.9%) when compared to rates for the U.S. population as a whole [1]. Pregnancy is an important developmental milestone that is associated with significant opportunities for weight gain [2] including carrying excess weight into a pregnancy or failing to lose weight gained during pregnancy. This excess weight associated with the childbearing years may contribute to obesity-related risk and illness later in life [3–8]. Physical activity (PA) has well-established beneficial effects on weight management. There is strong evidence that engaging in moderate-intensity PA most days of the week can improve health outcomes [3, 6–11].

Sedentary behavior is more prevalent among Latinas than among their Anglo counterparts, contributing to a relatively higher risk of poor health outcomes [1, 12]. While limiting energy intake plays an important role in decreasing one’s risk of obesity, increasing PA is an important strategy that has been successfully used to manage weight across the lifespan. Despite the known benefits of PA, 67.5% of Hispanic women of Mexican heritage of all ages fail to meet the 2008 Physical Activity Guidelines for 150 minutes/week of moderate-to-vigorous intensity activity, and 46.7% are classified as inactive [13]. Further, the problem of postpartum weight management is coupled with limited understanding of overweight, obesity, and PA with subsequent risk for conditions that may lead to chronic illness, mediated by unfavorable metabolic changes such as increased inflammatory processes.

Among Latinas, social support and strong peer exercise norms are consistently and positively related to PA. Social support is the most commonly reported correlate of PA for Latinas [14–18], and support can be an important mechanism for behavior change related to weight management [1–9]. Pregnant and postpartum Mexican-born Latinas view social support more essential to the maintenance of PA to a greater extent than women of other ethnic groups [19]. In data from the Women’s Cardiovascular Health Network, correlates of PA among Latinas included knowing and observing others who engaged in PA [15, 19–21]. An important correlate is the neighborhood environment that can promote or deter the desire to be physically active, but neighborhood characteristics such as safety concerns, heavy street traffic, and presence of stray dogs [22] may create barriers to regular PA. Neighborhood characteristics and resident perceptions about neighborhoods are associated with neighborhood-level socioeconomic status, obesity/body mass index (BMI) and related behaviors [23]. Factors associated with a neighborhood that can discourage healthy behaviors (e.g., healthy eating, PA) include unlighted streets, lack of curbs or sidewalks, limited neighborhood food purchase accessibility, and crime [24, 25]. The socioeconomic barriers to residing in more desirable areas tend to be higher for Latinos of Mexican and Puerto Rican origin than for other racial/ethnic groups [26], and living in more disadvantaged neighborhoods is associated with higher BMI values [23, 24, 27, 28].

Neighborhood characteristics are not the sole factors inhibiting PA participation as childbearing provides its own unique challenges. Women tend to decrease PA during pregnancy and after birth for many reasons, such as the demanding role transitions to new motherhood or the occurrence of depression symptoms [29]. Other barriers to PA after birth may be associated with cultural norms regarding the acceptability of PA or decreased opportunity to lose pregnancy-associated weight due to a shortened interconception phase.

Weight loss, especially fat loss, in the postpartum phase has important health consequences. Accumulating evidence from recent studies points to the role of proinflammatory cytokines released by fat tissue in generating the chronic inflammatory profile associated with obesity and its related metabolic disorders [30]. Obesity-associated insulin resistance is thought to result, at least in part, from chronic subclinical inflammation [31]. Rather alarmingly, this chronic subclinical inflammation is observed in both obese and overweight people [32]. Elevated concentrations of many circulating inflammatory factors are also considered markers of systemic inflammation, such as C-reactive protein (CRP), interleukin-6 and interleukin-8 (IL-6, IL-8, respectively), and plasminogen activator inhibitor 1 (PAI-1). These markers are associated with obesity and insulin resistance and also appear to predict the development of diabetes and/or cardiovascular disease*.* Fat tissue may contribute to the increased concentrations of inflammatory factors by producing endocrine and paracrine inflammatory factors (adipokines) [33]. Adipose tissue secretion of IL-6 may constitute up to a third of its plasma concentration [34]. The secretion rate of IL-8 by adipose tissue correlates with BMI [35] and may account for the elevated plasma IL-8 concentration found in obese people [36].

Regular physical activity suppresses tumor necrosis factor - alpha (TNF-α) production by fat tissue and thereby offers protection against TNF-α-induced insulin resistance [37]. Physical activity and diet, but not diet alone, decrease plasma levels of CRP and IL-6 and improve abdominal fat tissue metabolism [38]. As well, weight reduction, due to diet and physical activity, decrease circulating levels of CRP, IL-6, and IL-8 and decrease markers of fat tissue inflammation, such as the expression levels of IL-6, IL-8, and TNF-α in fat tissue [35]. In premenopausal women, changes in plasma concentrations of TNF-α correlate well with specific alterations in the relative amounts of subcutaneous fat mass and visceral fat mass after PA training [39]. One postulated mechanism by which PA and/or weight loss reduces circulating levels of the inflammatory markers is through a decrease in levels of cytokines produced by fat tissue [40]. For example, physical activity reduces the expression of IL-6 in fat tissue [35, 41] and increases circulating levels of anti-inflammatory cytokines, such as IL-1receptor antagonist (Ra) and IL-10 [37]. Improving the balance between pro- and anti-inflammatory markers may be a key factor underlying the metabolic benefits of PA [42], and for this study, we used (1) mRNA concentrations of representative inflammatory markers (IL-8, IL-6, and TNF-α) using Real Time PCR (RTPCR) and (2) protein concentrations of typical regulators of inflammation (NF-κb p65 and NF-κb Inducing Kinase).

Here we report the effects of *Madres para la Salud* (*Madres*), a theoretically driven social support intervention program, on health outcomes among postpartum Latina women. The study aims were (1) to examine the effectiveness of *Madres* in improving *distal outcomes* including (a) body fat, (b), waist circumference and waist-to-hip ratio, and (c) post-partum depression (PPD) symptoms among women enrolled in *Madres* as compared to an attention control group at 6 and 12 months post-intervention after controlling for dietary intake; (2) to examine the effectiveness of *Madres* in improving theoretical mediators (*proximal outcomes*) including (a) social support, (b) walking and other PA, and (c) energy intake; (3) to test whether PA is related to body fat and systemic and fat tissue inflammation; and (4) to test whether perceived neighborhood characteristics moderate the effects of the intervention on walking.

The study protocol was approved by the lead investigator’s institutional review board (IRB) and the IRB of the partnering medical center. Written consent was obtained from each participant.

## Methods

### Design

This study used a prospective, randomized, controlled experimental design with assessments prior to and following the intervention. Participants were assessed at baseline, 6, and 12 months after initiation of the intervention. In addition to testing the effectiveness of a walking intervention for a high-risk population via this randomized control trial (RCT), we examined the impact of number of minutes per week walked on outcomes. The study was approved ethically by the Office of Research Integrity and Assurance: Internal Review Board at Arizona State University, and Maricopa Integrated Health System Protection of Human Subjects review committee.

#### Setting

Community settings in a large Southwestern U.S. city were used for recruitment and data collection. In this area, 46.3% of the population identifies as Latino, the majority whom are of Mexican origin [43]. Recruitment settings included health fairs, Women, Infants, and Children (WIC) clinics, Early Head Start centers, a community center, a community health clinic, a postpartum class from a large medical center, churches with large proportions of Latino congregants, and Latino markets.

### Sample

Inclusion criteria were: (a) habitually sedentary (<2.5 hours of moderate-intensity PA a week) but able to participate in moderate-intensity walking, (b) self-identified as Latina, (c) 18 to 40 years of age, (d) 6 weeks to 6 months post birth, and (e) BMI of 25 to 35 kg/m2. Exclusion criteria were: (a) severe musculoskeletal or cardiorespiratory problems that would preclude participating in PA, (b) currently pregnant or plans to become pregnant within the next 12 months, (c) current use of antidepressants, anticoagulants, or herbal remedies that effect coagulation, (d) infectious illness, acute or chronic systemic inflammation, (e) regularly taking high doses of oral steroid medication, or (f) osteoporosis at baseline (bone mineral density ≥ 2.5 SD below the average for this age group).

**Figure 1:**
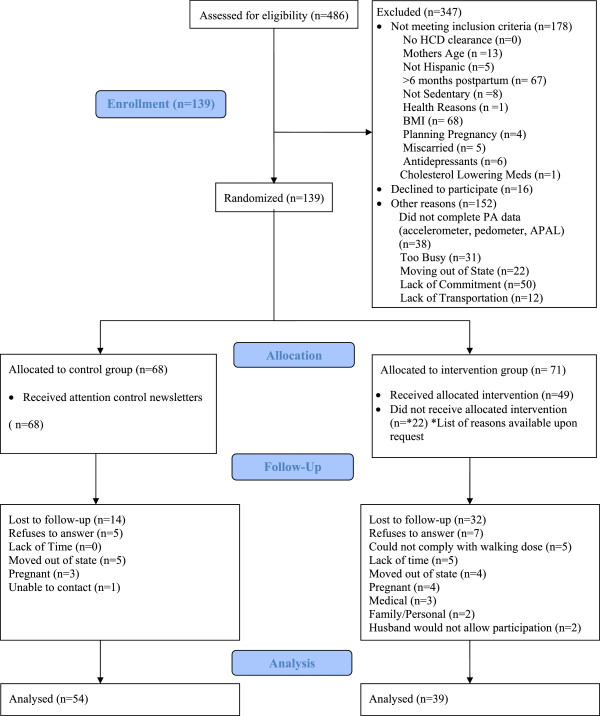
**Madres para la Salud CONSORT flow diagram.**

We enrolled 177 postpartum Latinas based on a power analysis and projected 50% attrition rate. After losing 38 women between enrollment and baseline data collection, 139 women were randomized to either the intervention (*n* = 71) or the control groups (*n* = 68). At the completion of the 12-month study, 93 participants remained (*n* = 39 intervention, *n* = 53 control) for an overall attrition rate of 33.81%. See CONSORT diagram (Figure 1).

### Procedures

*Madres* was a 12-month RCT designed to explore the effectiveness of social support in increasing PA to effect changes in body fat, fat tissue inflammation, and depression symptoms in sedentary postpartum Latinas who were obese or overweight. On the same schedule, the attention control group received monthly newsletters with information about health issues that were not related to PA, such as breast self-examination, diet, and emotional support related to new parenthood. The content given to the attention-control group did not include the “active ingredients” of the intervention but did include weekly telephone contact to answer questions about common postpartum or newborn concerns, such as breastfeeding, infant sleep, and sibling rivalry. Study materials, scales, participant instructions were delivered by bi-lingual, bicultural research assistants in the participant’s preferred language, and written materials were translated and back translated into Spanish.

The *Madres* intervention was conducted in a group format and led by trained *promotoras.* Detailed descriptions of the *Madres para la Salud* study design, measures, intervention components, and treatment fidelity are published elsewhere [44–46]. The intervention included doses of four different types of support (e.g., emotional, instrumental, appraisal, and informational) with group walking and intervention sessions held weekly over 12 weeks. A subsample of confirmed non-pregnant participants in the intervention group (*n* = 22) volunteered for fat tissue biopsy for inflammatory marker analysis, 16 of whom completed this procedure.

### Measures

The data reported here came from self-report paper and pencil questionnaires, electronic physical activity monitors (accelerometers and pedometers), archival data sources (U.S. Census and Phoenix Police Department reports), structured interviews, and anthropometric measurements as described below.

#### Participant demographic and background characteristics

Background variables included: (a) age in calendar years, (b) number of years of schooling completed, (c) socioeconomic status, measured as annual household income and number of individuals living in the household, (d) employment status and occupation, (e) number of pregnancies and number of births, (f) number of children living in the household, (g) weight before last pregnancy, (h) self-reported history of depression, (i) number of years in the United States, and (j) neighborhood supports for PA and healthful eating.

### Distal outcomes

#### Body composition

At each time point, waist and hip circumferences (in cm) were measured on each participant three times and averaged for that time point. Body Mass Index (BMI) was computed as weight in kilograms divided by the square of height in meters. We determined percent body fat via bioelectric impedance (BIA) using a portable four-terminal BIA measurement system (Tanita Corporation of America, Inc, Arlington Heights, IL) and methods outlined by Ritchie, Miller, and Smiciklas-Wright [59].

#### Depression symptoms

We assessed postpartum occurrence of depression symptoms using the Edinburgh Postnatal Depression Scale (EPDS), a 10-item, self-report measure capturing feelings of anhedonia, anxiety, sadness, and thoughts of self-harm over the previous 7 days. Response options ranged from 0 (e.g., *no, not at all, never*) to 3 (e.g., *yes, most of the time*; *yes, quite often*). Total scale scores range from 0 to 30, with higher scores indicating more severe depression symptoms. For this study, total scores ≥ 12 indicated the likelihood of depression and scores ≥ 16 indicated the likelihood of major depression. Cronbach’s alpha in the current sample ranged from .82 to .87 across the three time points. Women were referred to study physician (Coonrod) if any reported a “yes” answer to suicide ideation or reported a EPDS score >13.

#### Fat tissue inflammation

Subcutaneous fat biopsy was obtained from a subset of intervention-enrolled participants (*n* = 16) pre-intervention, and of these, only seven opted to have a repeat fat biopsy post-intervention. The tissue samples were immediately frozen after biopsy for subsequent RNA and protein extractions, using Trizol (Invitrogen, Grand Island, NY) and Nuclear Factor kappa b (NF-κb) Lysis Buffer (Active Motif, Carlsbad, CA) respectively. The RNA and protein extracts were assessed for inflammatory markers. The assessment consists of measuring (1) mRNA concentrations of representative inflammatory markers (IL-8, IL-6, and TNF-α) using Real Time PCR (RTPCR) and (2) protein concentrations of typical regulators of inflammation NF-κb p65 and NF-κb Inducing Kinase (NIK) using Western blot. Primers sequences for RTPCR (Sigma Genosys, The Woodlands, TX) and detailed description of antibodies for Western blot (Cell Signaling, Danvers, MA) are available upon request. The RTPCR was carried out using iCycler (Biorad, Hercules, CA). Western Blot gels were run using Invitrogen system (Invitrogen). Protein bands on Western Blots were visualized using Western Lightning™ Chemiluminescence Reagent *Plus* (Perkin Elmer, Waltham, MA) and quantified using FluorChem® Q (Alpha Innotech Corporation, San Leandro, CA).

### Mediators (Proximal Outcomes)

#### Social support for exercise

We used a 9-item self-report questionnaire measure adapted from the Social Support and Exercise Survey [47] to assesses the frequency with which family members and friends engaged in support of the respondents’ PA (e.g., “gave me helpful reminders to exercise”) and participated in exercise with the respondent (e.g., “exercised with me”). Response options ranged from 1 (*never*) to 3 (*often*). Social support for exercise has been related to reported current PA habits (*r* = 0.35–0.46) [47]. Cronbach’s alphas in the current sample ranged from .88 to .92 across the three time points.

#### General social support

We used the 19-item self-reported Medical Outcomes Study: Social Support Scale (MOS-SSS) [48] to assess how frequently social support was available in four domains: affectionate (3 items), tangible (4 items), emotional and informational (8 items), and positive social interaction (3 items). Response options range from 1 (*none of the time*) to 5 (*all of the time*). We computed a composite score for each domain and an overall score based on the summation of all 19 MOS-SSS items (which includes one item that is not part of any of the four MOS-SSS subscales). In previous work, in English- and Spanish-speaking samples, reliability coefficients for the four domain-specific composites were > .83 [49]. In the current study, the Cronbach’s alphas for domain-specific composites and the overall MOS composite ranged from 0.82 to 0.96 across all time points.

#### Self-reported physical activity

The Stanford Brief Activity Survey (SBAS) was used as a brief self-report screening tool and as a categorical measure of PA status [50, 51]. The SBAS uses reports of occupational (employment activity such as waitressing) and leisure-time PA (such as walking, tennis, or jogging) to classify respondents’ overall PA intensity levels on a 5-point scale as follows: inactive (1), light (2), moderate (3), hard (4), and very hard (5). Concurrent validity at T3 showed significant differences between SBAS categories with pedometer aerobic steps (F3,85 = 6.01, *p = .*0009) and time spent in aerobic walking, F (3, 85) = 5.59, *p = .*0015. Test-retest reliability showed modest associations between T3 and T5 administrations (weighted kappa = 0.32, 95% CI 0.17, 0.47).

#### Pedometer-measured physical activity

The Omron HJ-720ITC pedometer (Shelton, CT) is a dual-axis acceleration sensor that counts total steps, aerobic intensity steps, and distance walked. Aerobic intensity steps were determined using a counter mechanism that identifies >100 steps per minute as aerobic steps. The number of minutes spent in aerobic steps also was recorded.

Both the accelerometer and the pedometer were worn concurrently on opposite hips for 7 days during waking hours and at the same time the PA records were kept (e.g., baseline and 6 and 12 months). The minimal wear time for the ActiGraphaccelerometer for use in data analysis was 10 hours per day which is accepted as a best practice for assessing daily physical activity duration and intensity [52].

The accelerometer data were averaged over 7 days and are presented as minutes/day for sedentary, light, moderate-lifestyle, moderate-walking, and vigorous intensity. Total steps/day, aerobic steps/day, and aerobic steps minutes/day were obtained from the pedometer and averaged over 7 days.

#### Accelerometer-measured physical activity

PA duration at varying intensities was also assessed using data from the ActiGraph GT1M accelerometer (Pensacola, FL), a small, lightweight, biaxial accelerometer designed to measure the rate and magnitude of body movement in a vertical plane. Intensity values are presented as counts, with higher counts reflecting more intense movement. Using a one-minute epoch duration to aggregate movement counts, the Freedson [53] and Matthews [54] ActiGraph cut points were used to compute PA intensity levels as sedentary (<100 counts), light (100-760 counts), moderate-lifestyle (760-1,951 counts), moderate-walking (1,952-5,724 counts), and vigorous (>5,725 counts). Data are presented as the minutes at each intensity level.

A detailed protocol for the accelerometer used is described elsewhere [55], but briefly, participants were instructed to wear the ActiGraph for 7 days each at baseline, 6 months, and 12 months during the study. Each day they wore the monitor, participants were instructed to write the time they put the monitor on in the morning, when they took the monitor off before going to bed, and other times when they did not wear the monitor (e.g., bathing, swimming) in a record book. Data were included for analyses with counts recorded for 3 or more days with ≥10 hours/day.

### Moderator and covariate measures

#### Participant perceptions of neighborhood environment

We used a 33-item self-report instrument, comprising 7 subscales, adapted from the Neighborhood Environment Questionnaire [56] to assess respondents’ perceptions of their neighborhood environment including conduciveness to walking and PA, aesthetic quality, safety, access to healthy foods, neighbors’ engagement in activities with each other, social cohesion, and violence. In this sample, Cronbach’s alphas ranged from .69 to .87 across the 7 subscales.

#### Energy intake

Dietary intake data were obtained at baseline, 6 months, and 12 months using a 5-step, multiple-pass 24-hour dietary recall interview [57, 58] conducted by trained research assistants on two week days and one weekend day of the same week. During these unannounced interviews, respondents were asked to recall and report all foods and beverages consumed in the preceding 24 hours. The dietary data were analyzed using Nutrition Data System for Research software Version 2009 (Nutrition Coordinating Center [NCC], University of Minnesota, Minneapolis, MN), from which total energy intake and the proportion of energy from macronutrients were calculated.

### Analytic approach

The general approach we used in modeling changes in distal outcomes and hypothesized mediators (proximal outcomes) followed a 2 x 3 Group (Intervention vs. Control) x Time (repeated measures taken at baseline (T1), 6 months (T3), and 12 months (T5) analysis of variance (ANOVA) framework. In models predicting change in body composition measures, we treated energy intake, measured in kcal per day, as a covariate. Tests of overall main effects and the Group × Time interaction were followed by planned contrasts among cell means, testing (1) group differences at each time point and (2) group differences in the degree of change from (a) baseline to 6 months, (b) 6 months to 12 months, and (c) from baseline to 12 months. Associations between PA measures and inflammation markers were examined using Pearson correlations. Next, for models predicting PA, we elaborated on the basic 2 × 3 ANOVA framework described above by including one of seven continuous neighborhood environment measures (e.g., perceived safety) as a potential moderator of intervention effects on PA, resulting in 7 models (1 per moderator) for each of the 9 PA measures.

## Results

### Sample characteristics

The mean age of the women was 28.3 years (*SD* = 5.59). Most women were unemployed or never employed (*n* = 106, 75.8%) as compared with full or part time employment (*n* = 33, 23.9%). Type of employment reported included babysitter, cashier, cashier stocker, computer analyst, cook, manager, medical interpreter, vegetable packing, and waitress. This was the first pregnancy for 28 women (20.1%) with the remainder (*n* = 111, 79.9%) reporting 2-6 live births. Most of the participants were born in Mexico (*n* = 121), 1 was from Central America, and 19 were born in the United States. For those coming from outside the United States, years in the country ranged from 1 to 12 years; about half (48%) had resided in the country less than 10 years. Thirty-nine (27.9%) of the women had 1-2 children under the age of 2 at home, and 51 (36.5%) had 1-2 children aged 3-5 years at home. The majority of the participants (69.3%) reported household incomes of $20,000 or less. Characteristics of the subset of participants who underwent fat tissue biopsy are summarized in Table 1.

**Table 1:** **Baseline characteristics of subsample receiving fat biopsy (n = 16)**

Variable	***M***	***SD***
Age (years)	28.4	5.4
Body mass index (kg/m^2^)	29.5	2.8
Body fat (%)	38.7	3.7
ActiGraph wear time (min/day)	853.4	137.7
Sedentary time (% of total minutes)	58.2	9.8
Light PA (% of total minutes)	39.3	9.2
Moderate PA (% of total minutes)	2.4	2.1
Vigorous PA (% of total minutes)	0.0	0.0
Fat tissue IL-6 mRNA levels (arbitrary units)	3.8	6.8
Fat tissue IL-8 mRNA levels (arbitrary units)	1.7	1.6
Fat tissue TNF-α mRNA levels (arbitrary units)	0.9	0.2

American Community Survey data for 2007-2009 [43]showed that in the five ZIP Code areas in which our participants resided, the majority of residents were Hispanic or Latino (81.6%), spoke a language other than English at home (71.5%), and were not U.S. citizens (87.2%). A significant proportion were foreign born (38%), aged 25 and older with less than a 9th-grade education (25.3%), and families with children under the age of 18 who had income below the federal poverty level in the last 12 months (28.5%). Fourteen percent of housing units were vacant, 26% of residents lived in multi-unit housing structures, and 48.8% of households paid 35% or more in gross rent as a percentage of household income. Access to health care in these areas was limited, with 41.3% of residents having public health insurance and 34.7% of residents having no health insurance. City of Phoenix Police Department data (Phoenix Police Department) indicated these ZIP Code areas had the greatest number of incidents of domestic violence, homicides and robberies, aggravated assaults, drug crimes, and total violent crimes compared to other ZIP Code areas in the general urban area. These neighborhoods were second highest in the city for calls for service to the city’s police department, sexual assaults, total property crimes, and gang-involved incidents.

### Tests of intervention effects: distal outcomes

#### Percent body fat, waist circumference, and waist-to-hip ratio

As shown in Table 2, there was a significant Group × Time effect in predicting percent body fat, as measured by BIA, with controls showing a monotonic decrease over time and the intervention group showing a slight decrease from baseline to 6 months and then a slight increase from 6 months to 12 months. This pattern yielded a significant difference in the change from 6 months to 12 months (*p* = .0046). No other intervention effects were detected, but waist-to-hip ratio and waist circumference both decreased slightly from baseline to 12 months (*p* < .0001).

**Table 2:** **Unadjusted means and (Standard Deviations) by group and time effects on distal outcomes and mediators**

	Baseline	6 months	12 months		***p***-values	
Variable	***M (SD)***	***M (SD)***	***M (SD)***	Group	Time	Group x time
Body fat (%)^a^						
Attention control	38.55 (5.25)	38.19 (6.05)	37.72 (5.36)	.9645	.0267	.0170
Intervention	38.57 (3.98)	37.34 (4.88)	37.97 (4.37)			
Waist-to-hip ratio^a^						
Attention control	0.83 (0.07)	0.81 (0.05)	0.79 (0.05)	.1662	<.0001	.4177
Intervention	0.81 (0.07)	0.80 (0.06)	0.79 (0.06)			
Weight (kg)^a^						
Attention control	73.19 (10.40)	72.68 (11.52)	72.54 (11.30)	.6092	.1173	.6129
Intervention	73.75 (9.50)	71.82 (9.97)	71.99 (10.38)			
Depression^a^						
Attention control	8.69 (4.71)	7.80 (5.05)	6.98 (4.29)	.6372	.0335	.8361
Intervention	8.21 (5.22)	7.05 (5.36)	7.00 (5.69)			
Social support for exercise^a^						
Attention control	1.73 (0.53)	1.81 (0.55)	1.83 (0.57)	.4938	.0022	.0037
Intervention	1.75 (0.54)	2.05 (0.51)	1.72 (0.53)			
MOS Emotional/informational^a^						
Attention control	3.95 (0.87)	3.95 (0.93)	4.09 (0.90)	.0369	.4302	.3627
Intervention	3.81 (1.09)	3.56 (1.12)	3.69 (1.10)			
MOS Tangible^a^						
Attention control	3.68 (1.07)	3.72 (1.02)	3.70 (1.02)	.0523	.2240	.1825
Intervention	3.61 (1.17)	3.19 (1.17)	3.29 (1.14)			
MOS Affectionate^a^						
Attention control	4.34 (0.84)	4.35 (0.90)	4.46 (0.81)	.1926	.6302	.7454
Intervention	4.24 (0.89)	4.17 (0.98)	4.27 (0.88)			
MOS Positive social interactions^a^						
Attention control	4.00 (0.91)	4.01 (0.85)	4.12 (0.92)	.4282	.3800	.3857
Intervention	4.06 (1.04)	3.81 (0.96)	3.93 (0.95)			
MOS Overall^a^						
Attention control	3.95 (0.81)	3.97 (0.86)	4.06 (0.84)	.0636	.4512	.2854
Intervention	3.87 (0.98)	3.61 (0.99)	3.73 (0.95)			
SBAS^a^						
Attention control	1.60 (0.70)	1.78 (0.71)	2.06 (0.78)	<.0001	<.0001	<.0001
Intervention	1.58 (0.67)	2.63 (0.94)	2.82 (0.98)			
Total steps^b^						
Attention control	4909.74 (2255.70)	6040.16 (2561.94)	6425.04 (3390.90)	.0436	<.0001	.0177
Intervention	5120.33 (2244.01)	7575.60 (2785.47)	6963.91 (3126.54)			
Aerobic steps^b^						
Attention control	369.64 (646.03)	553.55 (964.98)	648.66 (1154.26)	<.0001	<.0001	<.0001
Intervention	447.36 (881.15)	2254.20 (1745.95)	1441.26 (1488.53)			
Aerobic walking time^c^						
Attention control	3.59 (6.27)	5.22 (9.13)	5.92 (10.03)	<.0001	<.0001	<.0001
Intervention	4.15 (7.75)	19.52 (14.97)	12.72 (13.19)			
Sedentary (no/low activity)^c^						
Attention control	505.00 (170.19)	468.55 (147.12)	463.09 (115.16)	.6478	.0951	.9521
Intervention	509.38 (159.08)	475.44 (154.97)	474.97 (123.05)			
Light lifestyle activity^c^						
Attention control	248.53 (53.76)	255.99 (66.76)	278.35 (76.52)	.3194	<.0001	.9125
Intervention	235.77 (47.18)	241.75 (49.43)	271.08 (79.14)			
Moderate lifestyle activity^c^						
Attention control	77.29 (46.25)	90.64 (49.52)	104.82 (52.84)	.0714	<.0001	.1505
Intervention	77.29 (35.32)	102.21 (45.46)	124.62 (66.90)			
Moderate walking activity^c^						
Attention control	15.33 (12.28)	20.62 (16.87)	23.61 (23.76)	.0021	<.0001	.0239
Intervention	17.67 (14.94)	34.18 (24.62)	38.97 (38.10)			
Vigorous activity^c^						
Attention control	0.16 (0.64)	0.66 (2.38)	0.39 (1.33)	.1627	.0713	.8818
Intervention	0.57 (1.95)	1.01 (3.43)	0.63 (1.71)			

^a^ *p*-values for Group effects from *F*-tests with 1 and 137 degrees of freedom; *p*s for Time and Group x Time effects from *F*-tests with 2 and 137 degrees of freedom. ^b^Steps/day; *p*-values for Group effects from F-tests with 1 and 135 degrees of freedom; *p*s for Time and Group x Time effects from *F*-tests with 2 and 135 degrees of freedom. ^c^Minutes/day; *p*-values for Group effects from F-tests with 1 and 136 degrees of freedom; *p*s for Time and Group x Time effects from *F*-tests with 2 and 136 degrees of freedom.

#### Depression

We found no Group × Time effect on EPDS scores, but we did find that scores significantly decreased for both groups from baseline to 12 months (F2,137 = 3.34, *p* = .0384). No effects were found on likelihood of major depression (EPDS score ≥ 16).

### Tests of intervention effects: mediators (Proximal Outcomes)

#### Social support

We found a significant Group × Time interaction in predicting Social Support for Exercise (SSE). Planned contrasts showed significant Group differences at 6 months (*p* = .0274), with the intervention group having higher levels of SSE than the control group. The interaction contrasts comparing change in SSE from 6 months to 12 months across groups was significant (*p* = .0013) with change being negligible in the control group and negative in the intervention group, such that SSE in the intervention group declined to levels seen at baseline.

We found no significant Group × Time effects on measures of general social support. Controls reported higher levels of emotional/informational support than did intervention participants overall (*p* = .0369), and specifically at the 12-month follow-up (*p* = .0400). Controls also reported higher levels of tangible support at 6 months than did intervention participants (*p* = .0282). No other significant differences were detected for the general social support measures.

#### Physical activity

We found a significant Group × Time effect on number of aerobic walking steps from the pedometer (see Table 2). The intervention group showed significantly higher numbers of aerobic walking steps than controls at 6 months and 12 months (*p* < .0001 and *p = .*0074, respectively). The complex patterns of change yielded a significant contrast, with a much larger increase in aerobic steps from baseline to 6 months for the intervention group than for the control group (*p* < .0001), an increase in aerobic steps from 6 months to 12 months among controls with a *decrease* in the intervention group (*p = .*0032), and a stronger overall linear trend from baseline to 12 months for the intervention group as compared to the controls (*p = .*0202). A parallel pattern of results was seen for total walking steps.

There was significant Group × Time effect (see Table 2) for aerobic walking time measured by the pedometer. The intervention group had more aerobic walking time than did the control group at both 6 months and 12 months (*p* < .0001 and *p* = .0095, respectively). In a pattern similar to that for aerobic walking steps, contrasts for Group differences in changes in aerobic walking time were all significant, with the intervention group showing a stronger baseline to 6 months increase than controls (*p* < .0001), controls showing an increase from 6 months to 12 months while the intervention group showed a *decrease* (*p* = .0050), and the intervention group showing a stronger overall linear increase from 6 months to 12 months than the controls (*p* = .0242).

The ActiGraph accelerometer data revealed a significant Group × Time effect in minutes per day of moderate intensity walking activity (see Table 2), with the intervention group showing a greater increase in moderate intensity walking than the control group. The intervention group showed higher levels of moderate intensity walking than did controls at 6 months and 12 months (*p* = .0008 and *p* = .0207, respectively). The intervention group showed larger increases in moderate intensity walking than did controls from baseline to 6 months (*p* = .0069). The Group differences in moderate walking intensity changes from baseline to 12 months (*p* = .0579) and from 6 months to 12 months (F < 1.0) were not significant. No significant changes were observed for sedentary time, moderate-lifestyle, and vigorous-intensity PA minutes/day in either group.

Light-intensity activity and moderate-lifestyle PA showed significant changes across time points. These increases, however, did not differ in magnitude across groups. The Time effect on light activity in the base model was qualified by a significant Group × Time × Violence interaction (*p* = .0337), such that all participants showed steady increases in this type of PA, except for *Madres* participants reporting lower levels of Violence who showed a slight drop from baseline to 6 months before showing an increase from 6 months to 12 months that was comparable to that seen in other participants.

#### Associations between mediators and distal outcomes: fat tissue inflammation

Daily proportion of light PA was negatively correlated with sedentary time (r = -.98, *p* < .001). Fat tissue mRNA expression levels of IL-6, IL-8, and TNF-α were positively correlated with sedentary time (*r* = .47, *p* = .081; *r* = .70, *p* = .004; *r* = .55, *p* = .035, respectively) and inversely correlated with light PA (*r* = -.50, *p* = .052; *r* = -.74, *p* = .001; *r* = -.58, *p* = .021, respectively). The relative amount of total NF-κb p65 in fat tissue at T5, but not at T1, was inversely correlated with light PA (*r* = -.76, *p* = .047) and moderate PA (*r* = -.89, *p* = .008).

#### Moderators of intervention effects on walking: neighborhood influences

Of the 7 Neighborhood × Group × Time interactions tested for SBAS physical activity sores only one was significant. Here we present findings (a) for the base 2 × 3 (Group × Time) ANOVA model of SBAS and (b) for the one analysis in which the 3-way interaction effect was detected. In the base Group × Time analysis, we found a significant Group × Time interaction, F(2, 137) = 11.17, *p < .*0001. Contrasts showed significant Group differences at 6 months (F(1, 137) = 26.42, *p < .*0001), 12 months (F(1, 137) = 17.47, *p < .*0001), change from baseline to 6 months (F(1, 137) = 17.89, *p < .*0001), and change from baseline to 12 months (F(1, 137) = 14.59, *p = .*0002) with the intervention group showing higher SBAS scores at the two post intervention time points and larger degrees of change over time than controls.

The Group x Time effect was qualified by a significant interaction with the measure of perceived neighborhood violence (Violence) obtained from the Neighborhood Environment Questionnaire, F(2, 118) = 3.56, *p* = .0315. Examination of estimated means at low (1 *SD* below the mean), average (mean), and high (1 *SD* above the mean) levels of Violence, showed that at baseline, intervention participants reporting higher levels of Violence had higher SBAS scores than those reporting low or average levels of Violence, but that at 6 months, those reporting higher levels of Violence had lower SBAS scores than other Madres participants. At 12 months, SBAS scores did not differ across levels of Violence. Among controls, participants reporting low levels of Violence had higher SBAS scores than did participants reporting average or high levels of Violence. In this group, SBAS scores at 6 months and 12 months were unrelated to Violence. The pattern overall pattern of means was otherwise similar to that reported for the base model.

Of the 21 Neighborhood × Group × Time interactions tested for pedometer-assessed PA measures only one was significant. Total pedometer activity (number of steps) showed significant Group differences in change over time (F(2, 137) = 4.15, *p* = .0177) with intervention participants showing strong increases from baseline to 6 months and a modest decrease from 6 months to 12 months while controls showed more modest, but steady, increases over time. The significant Group × Time interaction on total steps/day was qualified by a significant interaction with Violence (F(2, 118) = 3.14, *p = .*0468), such that in the control group, total steps/day increased steadily from baseline to 12 months, with Violence consistently negatively related to total steps/day across all time points (i.e., main effects of Time and Violence), while in the intervention group, change in number of steps (particularly from 6 months to 12 months) was positively related to the level of Violence. Intervention participants in higher Violence areas showed relatively stronger increases from baseline to 6 months than those reporting average or low levels of Violence. Intervention participants reporting higher levels of Violence showed slight increases in PA from 6 months to 12 months, while those reporting average and low Violence levels showed modest decreases. Given the large number of related hypothesis tests performed, the significant interactions reported here should be interpreted with caution.

#### Energy intake

In Table 3 we show how, on average, over 50% of participants’ energy intake was provided by carbohydrates, about 16% was provided by protein, and about 30% was provided by fat, with a negligible contribution to total energy intake from alcohol (<0.5% of total energy). Saturated fat contributed to about 10% of total energy intake. Roughly, one out of every 4 kilocalories was provided by simple sugars. We found no significant Group × Time interaction effects and no Time main effects on energy intake or in the proportion of energy derived from each of the macronutrient sources (see Table 3). The sole between-group difference was that saturated fats provided a higher proportion of energy intake for those in the intervention group than for those in the control group (see Table 3). We found a Time effect on energy intake, indicating that participants in both groups reporting a reduction in energy intake over time (see Table 3). The magnitude of this change did not differ between groups.

**Table 3:** **Unadjusted means and (Standard Deviations) by group and time effects on total energy intake**

	Baseline	6-months	12-months		***p***-values ^a^	
Variable	***M (SD)***	***M (SD)***	***M (SD)***	Time	Group	Group × time
Energy (kcal/day)						
Attention control	1462 (552)	1362 (618)	1295 (389)	.0239	.6202	.6434
Intervention	1471 (505)	1385 (488)	1394 (515)			
Energy from carbohydrate (%)						
Attention control	53.6 (6.7)	52.0 (8.0)	53.9 (9.8)	.4712	.5600	.2871
Intervention	53.7 (9.6)	52.9 (7.2)	51.4 (8.3)			
Energy from protein (%)						
Attention control	16.1 (3.3)	16.6 (4.7)	15.9 (4.7)	.2766	.7346	.5873
Intervention	16.5 (4.8)	16.0 (3.2)	15.5 (3.9)			
Energy from fat (%)						
Attention control	30.3 (5.8)	30.1 (6.2)	29.8 (7.6)	.7241	.0612	.3276
Intervention	31.1 (7.3)	31.0 (6.9)	32.7 (6.6)			
Energy from saturated fat (%)						
Attention control	10.1 (2.3)	9.9 (2.4)	9.5 (2.5)	.6933	.0098	.1979
Intervention	10.6 (2.9)	10.3 (2.5)	11.0 (2.6)			
Energy from alcohol (%)						
Attention control	0.1 (0.5)	0.1 (0.4)	0.4 (1.6)	.3797	.5409	.8131
Intervention	0.3 (1.3)	0.2 (1.3)	0.4 (1.4)			
Energy from sugars (%)						
Attention control	25.6 (7.3)	27.6 (8.1)	26.7 (9.1)	.3254	.2756	.2672
Intervention	26.1 (6.9)	26.0 (7.0)	24.4 (8.3)			

^a^ *p*-values for Group effects from *F*-tests with 1 and 137 degrees of freedom; *p*s for Time and Group x Time effects from *F*-tests with 2 and 137 degrees of freedom.

## Discussion

The effectiveness of the Madres intervention was supported by increases in social support for exercise following delivery of the 48-week social support intervention and increases in pedometer steps, aerobic steps and ActiGraph-determined minutes of moderate-intensity minutes of walking following the intervention, with subsequent improvement in body fat parameters at intervention completion.

### Social support

The importance of perceived social support in the Latinas in this study is borne out in much of the research literature of Latino values and “characteristics.” For example, Keefe, Padilla, and Carlos’ [60] seminal work with California-based Mexican families showed great reliance on the “*compadrazgo”* system, with support from extended family kinship networks living close together. Other work has yielded similar findings among postpartum Latinas. Thornton and colleagues [61] indicated that husbands were a primary source of support in promoting healthy eating and regular PA and in-laws were considered a secondary source of support. Our data show that 76% (*n* = 107) of the women in this study lived in households with 4-7 occupants who were primarily family members. Household size might explain the finding that family presence was related to perceived support. However, the results show that while social support increased during the social support intervention assessed at 3 months, the support declined to pre-intervention levels at the end of the 48 weeks walking conclusion. Our interview data following the intervention indicated that the majority of the Latinas’ perceived support for walking was from family members, including husbands, mothers, siblings, and to some extent in-law relations. The support was emotional, advisory, and instrumental with provision of childcare and room and board. For women in the Madres intervention, it appears that social support was effective when it was “active” (first 3 months, during the social support intervention delivery) leading to the possibility that participation in the study contributed to improvement in social support and subsequent increases in PA.

### Physical activity

There were significant improvements in pedometer-determined aerobic steps, time spent walking at an aerobic pace, and total steps taken and in accelerometer-assessed time spent in moderate intensity walking activities due the intervention. At baseline, the wide range of overweight and obesity in the Latinas in this study was accompanied by a low and narrow range of PA as measured by self-report SBAS, pedometer steps, and accelerometer data [62]. Given that we excluded women who were physically active from the study, their sedentary behavior was not surprising, but still lower than expected. The increases in intentional PA in the intervention group provide evidence that the support offered during study participation as well as perceptions of most neighborhood characteristics can facilitate walking behaviors in young mothers with infant and sibling children. The young Latinas recruited for *Madres* began this intervention with higher levels of obesity and sedentary behavior than reported by survey data, setting the stage for improvement following the *Madres* social support intervention [1].

In research examining associations among acculturation, overweight and obesity, and sedentary behavior acculturation has been found to be strongly associated with obesity but less strongly associated with obesity-related behaviors such as diet and physical activity [63, 64]. For example, in a study of Mexican women, the highly acculturated had higher BMI, fat mass, fasting insulin, and diastolic blood pressure than low-acculturated women [65].

Many acculturation measures assess language use and comfort with the use of native language; however, emerging thought considers that other factors, such as length of time in the country and contextual nuances of neighborhood life, might contribute to sedentary behavior and obesity [66–68]. Research suggests that immigration status, length of residence in the U.S., socioeconomic status, and residential environment might play a much larger role in the health statue of culturally diverse and immigrant women than previously thought. Findings based on Latina members of focus groups who discussed their immigration status and weight gain/overweight showed that immigration affects cultural practices and may negatively influence diet quality through lack of preferred foods, social isolation, and food choices [69, 70]. Other research has found that Latinas who become more acculturated may be at greater risk for certain adverse health outcomes and engage in less PA [71]. As seen in our data, recent immigrants may move to neighborhoods where safety concerns make the areas less conducive to PA, thereby contributing to current or future obesity risks. These findings raise important questions regarding how immigrants make choices about relinquishing values and behaviors of their native cultures and adopting those of the mainstream U.S. culture and how these choices affect health and related behaviors as time living as a U.S. resident increases.

### Dietary intake

As compared to 2009-2010 population data on all women, dietary intake reported at baseline in this sample of young Latinas was lower than expected for total calories [72] and higher than expected for percentage of fat intake. This finding is consistent with data showing underreporting intake among Mexican American women [73], that individuals using 24-hour recall underreport energy intake by 16-20% [74], and our own earlier research showing a similar pattern of under-reporting or under-expected energy intake among Latinas [75]. Though the data are inconclusive, plausible reasons for under-reporting of energy intake include women forgetting where and with whom food was consumed; eating patterns geared to food and food preparation for growing families; or patterns in dietary intake in the context of gender, culture, and ethnicity that have been reported as significant confounders by others [75]. The joint influence of all or some of these factors on underreporting of consumption could explain the apparent paradox of low caloric intake coupled with overweight and obesity in this sample of Latinas.

### Neighborhood characteristics

Aspects of a neighborhood’s built environment that contribute to healthy behaviors (e.g. healthy eating and PA) include safety, lighted streets, curbs, neighborhood food purchase accessibility, and crime. Hispanics have been shown to be more socioeconomically limited in their ability to live in or move to better neighborhoods than other groups [76], and living in more disadvantaged neighborhoods may contribute to overweight and obesity [24]. Baseline data of the Neighborhood Environment Questionnaire showed that the Latinas in this study judged their neighborhoods to be somewhat below the mean in interest, shared values, availability of healthy foods, safety cohesion, and interaction with neighbors. They assessed violence as above average. Census and community survey data underscored the women’s modest neighborhood support and indicated that their residential milieus were suboptimal for engaging in neighborhood support and outdoor activities. The neighborhoods from which our sample was drawn were largely comprised of immigrants and were often targets of immigration checks by local law enforcement officers, resulting in behaviors that prioritized hidden identity (i.e. not walking in neighborhoods, shopping) behaviors that often precluded the neighborhood support and cohesion needed for optimal health.

### Parameters for body fat

The young women in this study showed parameters for body fat in the “obese” range, and declines in body adiposity assessed using bioelectric impedance. Because we did not obtain pre-pregnancy weight and the majority of the participants had several children, we are unable to assess if the weight postpartum was retained from earlier pregnancies or the most recent pregnancy. Nonetheless, overweight and obesity in this group of Latinas mirror those of Latinas in national surveys, indicating significant obesity-related health risks in these young women as a group [1]. The weight gained during pregnancy and retained at postpartum is often characteristic of central adiposity, and these deposits contribute to insulin resistance [5, 77]. Thus, the health risks associated with pregnancy weight gain and retention place these women at risk for future metabolic disorders.

What is interesting though is while the physical activity increased among the treatment group in terms of steps, aerobic steps and duration of aerobic steps, this had relatively little short-term impact on body fat, and no sustained effect on body fat. Indeed, the control group showed a monotonic decrease over time and the intervention group showing a slight decrease from baseline to 6 months and then a slight increase from 6 months to 12 months. This shows that for initial and sustained fat loss, physical activity needs to be sustained and aerobic, and certainly more effective with dietary moderation.

#### Fat tissue inflammation

While findings from a few studies (e.g., [78]) suggest that exercise reduces systemic inflammation associated with obesity, to our knowledge, this is the first study showing that inflammatory markers (IL-6, IL-8, and TNF-α) in fat tissue of overweight and obese women correlated positively with sedentary activity and negatively with light activity during normal daily life at baseline. Although the small sample size for the inflammatory marker group may have contributed to the overall lack of statistical significance when examining changes over time, light- and moderate-intensity activity after the intervention correlated negatively with fat tissue content of a common regulator in inflammation (NF-κb). The inflammatory markers chosen in this study served as representative, typical markers that have been shown to play important roles in linking fat tissue inflammation with systemic inflammation and subsequent metabolic disorders [79]. At best, the findings suggest that sustained moderate-intensity walking is needed to help reduce inflammation in fat tissue, and possibly other organs, thus minimizing systemic inflammation and related diseases.

#### Depression symptoms

The Madres intervention had no impact on depression rates among this group of postpartum Latinas, and depression symptoms showed a predicted decline across the 12-months of the study. The impact of mental health on physical health and associated morbidities is complex [80]. Some research shows that depression is high among Hispanic groups [81, 82] and higher among Mexican Americans and Puerto Ricans than among non-Hispanic whites [83–85], but that within Hispanic groups, rates of depression vary depending on socio-demographic, health, and economic factors [80, 81]. The Latinas in this study were at high risk for depressive symptoms after birth possibly due to their socioeconomic status, neighborhood characteristics, and primary language. Our choice to use higher cut scores for measurement of depressive symptoms may have resulted in women with problematic depressive symptoms being categorized as non-depressed (scores ≤12). Cut scores of 7/8 for likelihood of depression and 11/12 for likelihood of major depression have been used to evaluate postpartum women living in Mexico [86]. In a validation study, Lagerberg and [87] colleagues found a specificity of 24% when using the traditional cut score of 11/12 as we did in the present study. Sensitivity of 61% and specificity of 82% were found in the Lagerberg study when using cut scores of 6/7. Thus, a lower cut score than was used in this study may be warranted, especially when working with disadvantaged groups or those for whom English is not a first language.

## Conclusions

This study reports the effects of the *Madres para la Salud* program on health outcomes among postpartum Latinas and examined the effectiveness of the *Madres* intervention for improving (i.e., reducing) the distal outcomes of (a) percent body fat, (b) fat tissue inflammation, and (c) depression symptoms among postpartum Latinas and determined the relationship between the immediate outcome of walking (minutes walked per week) and change in the distal outcomes. While the social support intervention showed increases in social support, the link between these increases were only tenuously linked to increases in PA and body fat reduction. Some limitations are apparent in this protocol: 1) the attention control group was not an equal attention-control group- receiving only monthly newsletters, and 2) the use of the BIA as a measure of body fat is subject to variability of lean tissue, hydration and age. Three implications from this report are evident: 1) the behavioral intervention of social support was effective in increasing PA among the women in the intervention study arm, but only during active treatment delivery, 2) the dose of moderate PA without dietary intervention was insufficient to reduce body fat in the treatment arm, and 3) the possibility that neighborhood environment (i.e. safety), contributed to the lack of sustainability in women’s walking. Further research requires a combination of diet and PA to achieve weight (fat loss) and social support strategies need to be determined that achieve long lasting behavioral change in PA.
